# Characterization of Complete Mitochondrial Genome and Phylogenetic Analysis of a Nocturnal Wasps—*Provespa barthelemyi* (Hymenoptera: Vespidae)

**DOI:** 10.3390/cimb45120587

**Published:** 2023-11-22

**Authors:** Mandie Liu, Yifei Luo, Binta J. J. Jallow, Fanming Meng

**Affiliations:** College of Basic Medical Science, Central South University, Changsha 410017, China; liumandie20@163.com (M.L.); 216511064@csu.edu.cn (Y.L.); bintajjjallow@gmail.com (B.J.J.J.)

**Keywords:** mitochondrial genome, nocturnal wasps, Vespinae, phylogeny

## Abstract

Genus *Provespa* contains nocturnal wasps mainly found in the southeastern region of Asia. There are no complete genome resources available of this genus, which hinders the study of its phylogenetic evolution and the origin of nocturnal behavior in the Vespidae family. Through high-throughput sequencing, we obtained the mitochondrial genome of *Provespa barthelemyi* (Hymenoptera: Vespidae), which is 17,721 base pairs in length and contains 13 protein-coding genes (PCGs), 22 tRNAs, and two rRNAs. We identified four gene rearrangement events of *P. barthelemyi* that frequently occur in the Vespidae family. We used Maximum Likelihood (ML) methodologies to construct a phylogenetic tree based on the sequenced mitochondrial genome and the available data of reported species belonging to Vespinae. Our findings confirmed the monophyly of Vespinae. Our study reports the first complete mitochondrial genome of *Provespa* and compares its characteristics with other mitochondrial genomes in the family Vespidae. This research should shed light on the phylogenetic relationships and ecological characteristics of the Vespidae family.

## 1. Introduction

As an ancient insect group on Earth, Vespidae has a large population and wide distribution, which is closely related to human life. For one thing, wasps can eat fruit and trees, which has a negative effect on agricultural production [1]. Female wasps with toxic stings can cause allergies and even death, which is a hidden danger to human health [2,3]. On the other hand, wasps can feed on agricultural pests and pollinate crops, which make a great contribution to ecological balance [4,5,6]. It is of great significance to understand and study this family.

Containing the representative species of solitary, sub-sociality to true sociality and so on, the Vespidae is an ideal group to study the social evolution of insects [7,8,9,10]. However, the social evolutionary origin of the family Vespidae and the phylogenetic relationship in the subfamily and genus remain controversial [11,12,13,14]. The Euparagiinae typically leads a free-roaming lifestyle without fixed communal nests, exhibiting completely solitary behavior [15]. Masarinae resemble bees, with multiple females of individual species sharing the hive [16,17]. Eumeninae is a subfamily that has the largest population among Vespidae, and most species are solitary [18,19]. The members of Polistinae already have social characteristics, but there is almost no visible difference between queen bees and working bees [20,21]. Finally, it further evolved into the Vespinae, which has high sociality, a clear division of labor and a large number of members [7,22]. From these subfamilies, we can see the gradual evolution of the wasp family from solitary to social [23]. Carpenter largely supported a single origin of eusociality by studying the living habits and phylogenetic evolutionary relationships of six subfamilies of Vespidae, believing that the sociality of Euparagiinae was the lowest, while that of Vespinae was the highest [24,25]. However, in 1998, Schmitz and Moritz argued that the sociality of wasps underwent two independent evolutionary processes, with one branch being Stenogastrinae and the other being (Vespinae + Polistinae), which was established through nuclear 28S ribosomal DNA sequence analysis [26]. This is key to solving the phylogenetic problem of Vespidae by further studying the classification of this family and obtaining more representative species and characteristic data [27].

The genus *Provespa* is a primitive taxon in the family Vespidae and is important for the study of the social behavior and evolution of the various groups of the Vespidae [25]. Additionally, *Provespa* exhibit a remarkable nocturnal lifestyle [28]. They can be attracted to optical sources in field studies. Little information about the origin of such nocturnal behavior in the Vespidae family has been reported so far. *Provespa barthelemyi* (Hymenoptera: Vespidae) has a primary geographic range in Southeast Asia, extending from the Indian Peninsula across southern China to the northern part of the Malay Peninsula [29]. In the present paper, we sequenced and assembled the mitochondrial genome of *P. barthelemyi*. In addition to analyzing the sequence characteristics of the mitochondrial genome, we discussed the taxonomic status of *P. barthelemyi* in Vespidea from the mitochondrial genome level and analyzed the phylogenetic relationships among the subfamilies in combination with the reported complete mitochondrial genome sequence of wasps.

## 2. Materials and Methods

### 2.1. Sampling and DNA Isolation

The samples of *P. barthelemyi* were collected in the town of Lvchun, Yunnan Province, China, by using a light trap in the evening. After collection, all of the samples were immediately restored in pure ethanol for the subsequent chemical and molecular analyses. The voucher samples were restored at Central South University, Changsha, China. We used the DNeasy tissue kit (Qiagen, Beijing, China) to extract the DNA using the manufacturer’s recommended protocol.

### 2.2. Mitochondrial Genome Sequence Reading and Assemblage

A high-throughput sequencing technique was used to read the mitochondrial DNA sequences of *P. barthelemyi*. Illumina TruSeq library was constructed by taking 1 μg of purified genomic DNA (~500 bp) from each sample (Shanghai, China) using the TruSeq™ Nano DNA Sample Prep Kit (FC-121-4003). The sequencing was then accomplished using an Illumina NovaSeq 6000 platform with a 150 bp paired-end read length. Before assembly, Trimmomatic 0.39 was used to filter the raw reads [30]. Three procedures were used to reconstruct the mitochondrial genome. First, the pair-end reads were assembled into larger segments called contigs by overlapping using MitoZ v2.3. Potential mitochondrial contigs were extracted by aligning them against the NCBI mitogenome database [31]. Second, we used BLAST v 2.8.1+ to perform homologous gene alignment and aligned contigs (>80% query coverage) were ordered and connected through a manual comparison with the reference mitochondrial genomes [32,33]. Finally, MUMmer 3.23 was used to check whether the result of the assembly was circular. Based on the above assembly steps, we obtained a circle of the *P. barthelemyi* mitochondrial genome [34]. The sequenced mitochondrial of the *P. barthelemyi* was deposited in GenBank under accession number NC_079667.1.

### 2.3. Mitochondrial Genome Annotation and Characterization

The mitochondrial genes of *P. barthelemyi* were annotated (predict PCGs, tRNA genes, and rRNA gene), and secondary structure modeling was completed using the MITOS WebServer (http://mitos2.bioinf.uni-leipzig.de/index.py. Accessed on 2 March 2023) [35]. The BLAST search (https://blast.ncbi.nlm.nih.gov/Blast.cgi. Accessed on 5 March 2023) for the reference mitochondrial genome sequence was used to find the position of each gene on the mitochondrial genome circle. The start/stop codons were manually corrected in SnapGene Viewer. The circular mitochondrion genome map of *P. barthelemyi* was drawn using the Cgview tool (JavaScript version). The skew of A-T and G-C were performed as following these calculations: AT-skew is equal to the value of A minus T divided by the value of A plus T. Similarly, GC-skew is equal to the value of G minus C divided by the value of G plus C [36]. EMBOSS v6.6.0.0 was used to work out relative synonymous codon usage (RSCU) [37,38]. The phylogenetic relationship cladogram was generated via the maximum likelihood (ML) method using IQ-TREE (http://iqtree.cibiv.univie.ac.at/. Accessed on 20 March 2023). MEGA 11 was used to align the protein-coding genes (PCGs) [39]. Using ModelFinder to obtain the optimal evolutionary model of the target data quickly, ML analysis was performed with bootstrap confidence estimate repeated 1000 times [40,41]. FigTree v1.4.4 was used to edit and beautify the phylogenetic trees.

## 3. Results and Discussion

**Figure 1:**
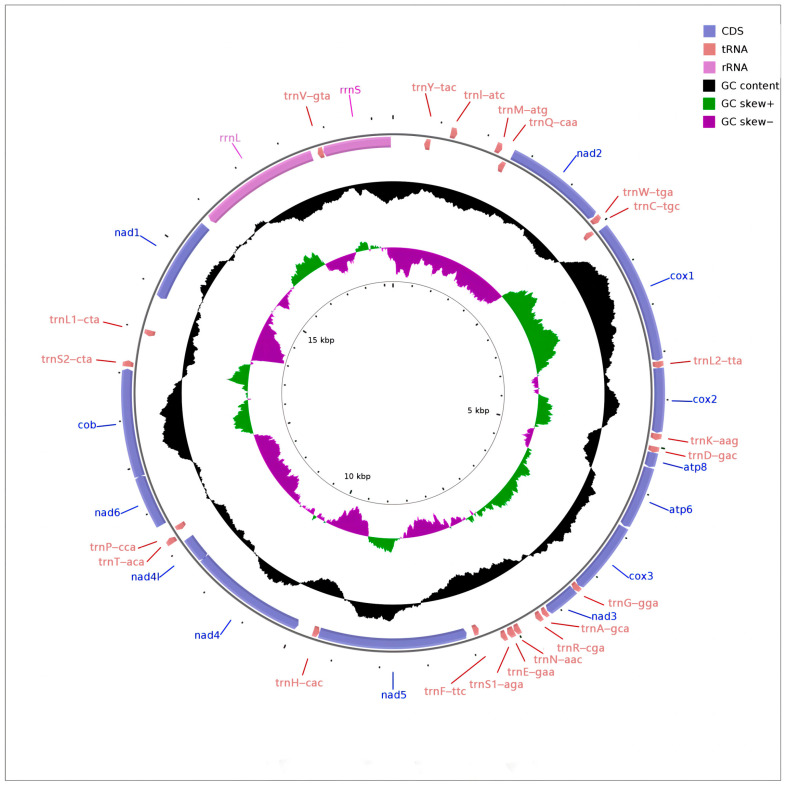
Circular genome map of *Prevesap barthelemyi* mitochondrial DNA.

The mitochondrial genome of *P. barthelemyi* (accession number NC_079667.1) encodes 13 PCGs, 22 tRNAs, and two rRNA genes and was 17,721 bp in length. There are 23 genes on the J-strand and 14 on the N-strand (Table 1) (Figure 1). The rearrangement events of *P. barthelemyi* mitochondrial genome reported in this study were basically consistent with the known events in the subfamily Vespinae, including the initiation of tRNA arrangement as trnY-trnI-trnM-trnQ, translocation of trnL1 to the region between trnS2 and nad1 and the shuffling of trnE and trnS1 [42,43,44,45,46,47].

The base composition of the mitochondrial genome was determined as follows: A = 38.53%, G = 5.18%, C = 14.84%, and T = 41.45%, with a GC content equal to 20.02% and an AT content equal to 79.98%, which is similar to other Hymenopteran mitogenomes [48]. Li et al. found that Stenogastrinae had the maximum A + T content value, while Vespinae had the lowest when sequencing the mitochondrial genome of Vespidae [44]. In Vespinae, *Dolichovespula* shows a high AT content, usually more than 80% [46]. The A-T skew (−0.037) and G-C skew (−0.483) of the *P. barthelemyi* genome were all negative, indicating that the percentage of T and C were higher than A and G in the sequence of the mitochondrial genome, respectively. A-T skew and G-C skew were both negative in PCGs, which were positive in tRNAs and rRNAs, showing that the content of T and C were higher than A and G in PCGs, respectively, whereas the opposite was true for tRNAs and rRNAs.

**Figure 2:**
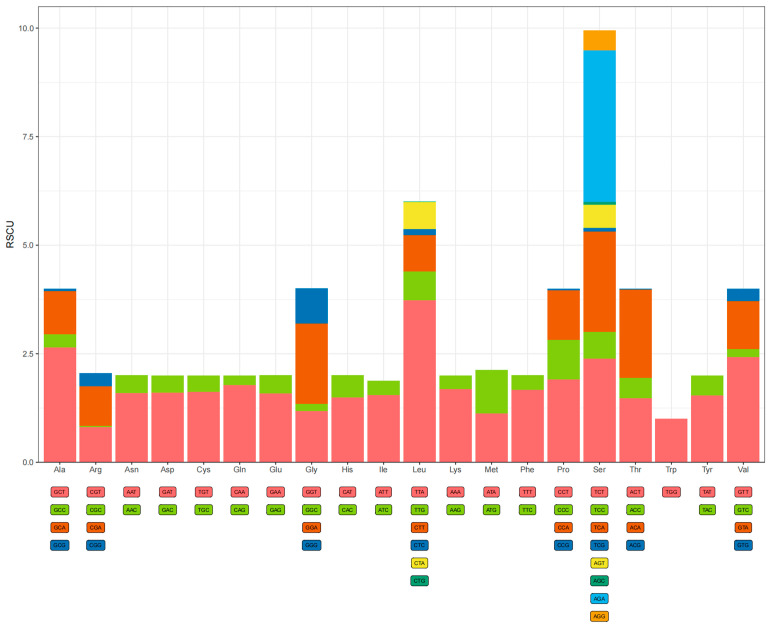
Relative synonymous codon usage (RSCU) in *Provespa barthelemyi*.

PCGs of the *P. barthelemyi* mitochondrial genome comprise a total of 3430 codons. Among the 13 PCGs of the mitochondrial genome, there were five start codons, namely ATT, ATG, ATA, ATC, and TTG. ATT is the most frequently used start codon (atp6, atp8, nad1, nad4l, and nad5), followed by ATG (nad2, nad4, nad6, and cox3), ATA (nad3 and cob), ATC (cox2), and TTG (cox1). Except for nad1 and cox3, which have TAG and T as the stop codons, respectively, the rest of the PCGs (cox1, cob, nad2, cox2, atp8, atp6, cox3, nad3, nad5, nad4, nad4l, and nad6) have TAA as the stop codons. RSCU and amino acid usage in the PCGs of *P. barthelemyi* are summed up in Figure 2. If a codon is without preference, the RSCU value of the codon is equal to 1. When the RSCU value of a codon is above 1, it means that the codon has relatively high use, and vice versa [49,50]. The three most common codons for the three amino acids were ATT (Ile), TTA (Leu), and TTT (Phe), with a codon usage of 376 bp and so on. The base compositions of the above codons were all A and T, indicating that the codons of the mitochondrial PCGs were biased toward bases A and T. The most frequently used codon was Leu (15. 4%), followed by Ile (12.1%) and Phe (10. 9%), which is also common in species of Vespidae [46]. Supplementary Table S2 provides specifics regarding codon usage. 

**Figure 3:**
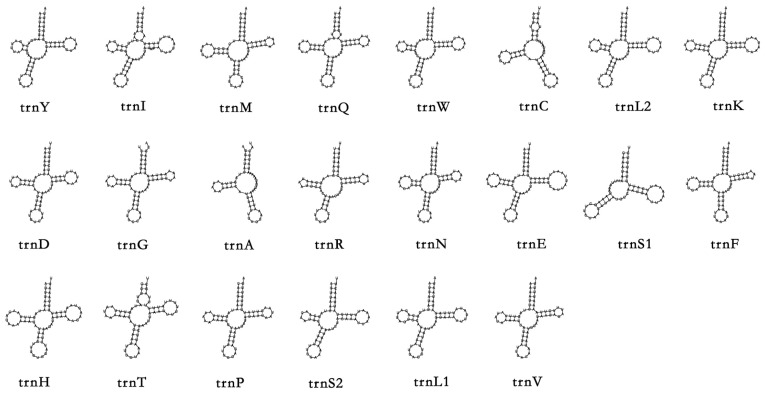
Selective pressure analysis in the protein-coding genes of *Provespa barthelemyi*.

In the mitochondrial genome of *P. barthelemyi*, the lengths of 22 tRNAs range from 75 bp to 57 bp. Nineteen of the twenty-two tRNA genes form typical cloverleaf secondary structures, except for trnC, trnA, and trnS1 (Figure 3). Both trnA and trnC lack the TψC (T) arm. The lack of TΨC loop of trnH has been observed in several species of *Dolichovespula* [51]. TrnS1 gene exhibited the deletion of the Anticodon (AC) arm. Other co-familiar wasps, such as *Parapolybia crocea* and *Orancistrocerus aterrimus aterrimus*, also presented an absent structure in the trnS1 gene. A truncated trnS1 gene represents a conserved mitochondrial feature in eumetazoans. Compared with other Vespidae, the duplication of trnM genes in the Eumeninae is relatively common.

**Figure 4:**
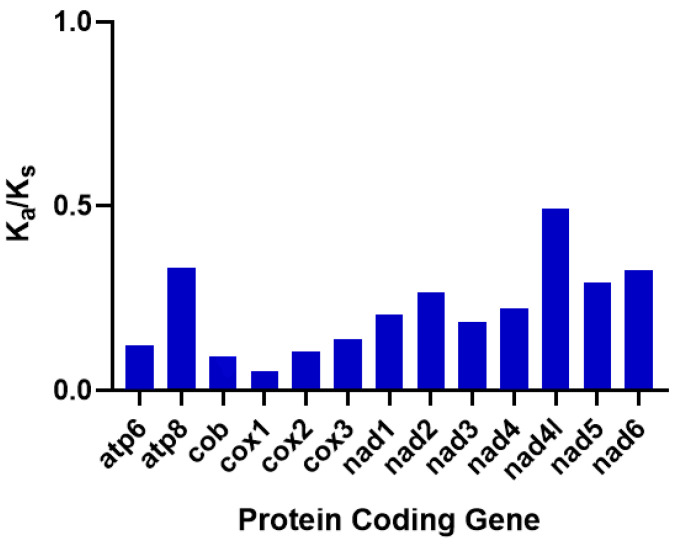
Secondary structures of 22 transfer RNA genes in *Provespa barthelemyi*.

To value the selective pressures working on *P. barthelemyi*, we calculated non-synonymous (Ka) and synonymous (Ks) substitution ratios (Ka/Ks) for all PCGs. In this study, 13 PCGs showed values well below 1, indicating that all genes undergo strong purifying selections (Figure 4) [51]. The nad4l has the highest Ka/Ks value (0.49), followed by atp8 and nad6, whose Ka/Ks value is 0.33 and 0.32, respectively, indicating that the selection pressure of these genes was relatively weak. It has been reported that four subfamilies (Stenogastrinae, Vespinae, Polistinae and Eumenidae) of the Vespidae also has high Ka/Ks value of nad4l based on PCGs [52]. The evolution of NADH genes is generally faster than that of cytochrome oxidase genes. Cox1 has the lowest Ka/Ks value (0.05), followed by atp8 and nad6, whose Ka/Ks values are 0.09 and 0.10, respectively, indicating that these genes are subjected to strong selective pressures. Recent studies show that the cox1 of the Vespidae and the subfamily Eumeninae has the lowest observed values, which suggests that cox1 is highly conservative, has functional stability and is suitable for molecular barcoding [44,53,54].

**Figure 5:**
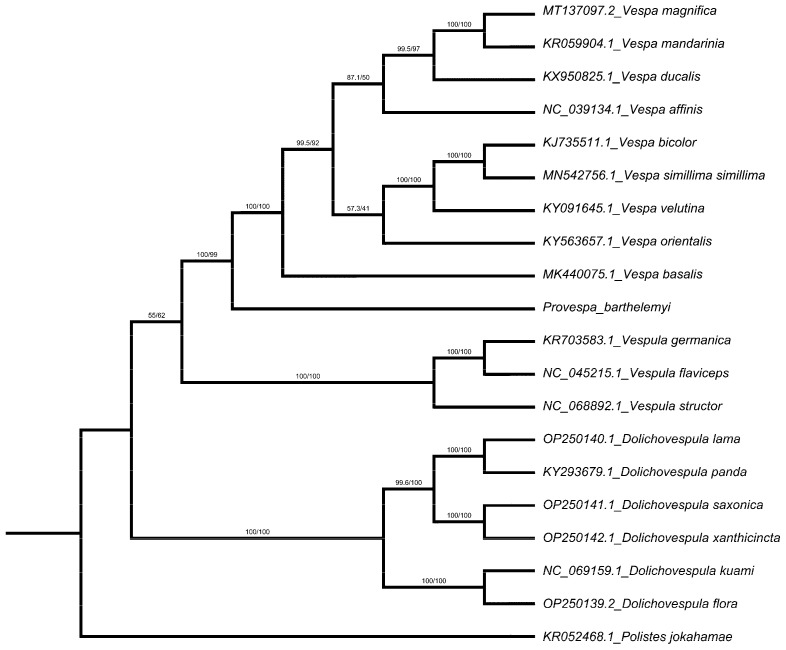
Construction of phylogenetic tree determined via maximum likelihood (ML) analysis of 13 PCGs based on *Provespa barthelemyi* and 19 species of the Vespinae, with one specie of the Polistinae as an outgroup. The number on the branches indicates the ML bootstrap supportvalue.

With *Polistes jokahamae* as an outgroup, the mitochondrial genomes available in the Genbank database belong to relationship analysis (Figure 5). Based on the nucleotide sequences of 13 PCGs, the optimal model predicted using ModelFinder was GTR + F + I + G4. The molecular evolutionary tree was created using the maximum likelihood method (ML), bootstrap values (bv) of 1000, with various representatives of Vespinae (20 sequences, 11,205 nucleotide sites, 4956 parsimony informative sites). Within Vespinae, the tree shows the relationship among genera is *Dolichovespula* + (*Vespula* + (*Provespa* + *Vespa*)), and the monophyly of the Vespinae is strongly supported. Based on the appearance and behavioral characteristics, the cladogram constructed by Carpenter showed that *Vespa* was the first clade to diverge, followed by *Provespa*, with *Dolichovespula* and *Vespula* grouped together [24]. Saito et al. believed that *Dolichovespula* and *Vespula* formed sister clades [29]. The two got together to form sister clades with *Provespa*, and *Vespa* is the sister group of the genera above. Based on nucleotide sequence analysis, the phylogenetic relationships of four genera identified by Perrard are as follows: *Dolichovespula* + (*Vespula* + (*Provespa* + *Vespa*)), but combining morphological and wing venation analysis, completely different phylogenetic results were obtained, indicating that there was a contradiction between morphological classification and molecular classification of Vespinae [55]. The results of Lopez-Osorio and Pickett’s combined analysis provide strong evidence for the hypothesis that *Dolichovespula* and *Vespula* are sister clades [51]. However, in another report, Osorio et al. concluded that *Dolichovespula* is more closely related to *Vespa* than to *Vespula* based on transcriptomic data without *Provespa* because sequencing data were not available [56]. Recently, some researchers found the phylogenetic relationships of Vespinae to be *Dolichovespula* + (*Vespula* + *Vespa*) using mitochondrial sequencing techniques [57,58,59,60], which is basically consistent with our study. Supplementary Tables S1 and S3 show detailed information for the species included and ML analysis.

## 4. Conclusions

In this study, the complete mitochondrial genome sequence of *P. barthelemyi* (accession number NC_079667.1) was sequenced and annotated for the first time, which enriched the mitochondrial genome sequence information of the Vespidae family and provided useful molecular data for molecular phylogenetic analysis. At the same time, the analysis of its structural characteristics and base composition provided basic data for the comparative analysis of mitochondrial genomics of those nocturnal species. Finally, phylogenetic tree analysis based on the nucleotide sequences of 13 PCGs from 19 species of Vespinae filled up the phylogenetic relationship of *Provespa* in Vespinae and also laid a preliminary molecular foundation for the establishment of subsequent phylogenetic relationships and taxonomic status of Vespidae.

## Figures and Tables

**Table 1:** Mitochondrial genome of *Provespa barthelemyi*. arrangement and annotation.

Gene	Type	Strand	Position (Start-End)	Length (bp)	Intergenic Spacer	Start	Stop	Anticodon
trnY-tac	tRNA	N	350–417	68	-	-	-	ATG
trnI-atc	tRNA	J	617–687	71	199	-	-	TAG
trnM-atg	tRNA	J	1121–1188	68	433	-	-	TAC
trnQ-caa	tRNA	N	1222–1289	68	33	-	-	GTT
nad2	pcg	J	1313–2398	1086	23	ATG	TAA	-
trnW-tga	tRNA	J	2410–2478	69	11	-	-	ACT
trnC-tgc	tRNA	N	2483–2545	63	4	-	-	ACG
cox1	pcg	J	2553–4088	1536	7	TTG	TAA	-
trnL2-tta	tRNA	J	4097–4166	70	8	-	-	AAT
cox2	pcg	J	4167–4853	687	0	ATC	TAA	-
trnK-aag	tRNA	J	4861–4930	70	7	-	-	TTC
trnD-gac	tRNA	J	4999–5065	67	68	-	-	CTG
atp8	pcg	J	5066–5224	159	0	ATT	TAA	-
atp6	pcg	J	5231–5902	672	6	ATT	TAA	--
cox3	pcg	J	5915–>6698	784	12	ATG	T	-
trnG-gga	tRNA	J	6699–6764	66	0	-	-	CCT
nad3	pcg	J	6765–7121	357	0	ATA	TAA	-
trnA-gca	tRNA	J	7131–7187	57	9	-	-	CGT
trnR-cga	tRNA	J	7204–7268	65	16	-	-	GCT
trnN-aac	tRNA	J	7468–7533	66	199	-	-	TTG
trnE-gaa	tRNA	J	7536–7610	75	2	-	-	CTT
trnS1-aga	tRNA	J	7628–7688	61	17	-	-	TCT
trnF-ttc	tRNA	N	7879–7945	67	190	-	-	AAG
nad5	pcg	N	8022–9701	1680	76	ATT	TAA	-
trnH-cac	tRNA	N	9702–9773	72	0	-	-	GTG
nad4	pcg	N	9954–11,267	1314	180	ATG	TAA	-
nad4l	pcg	N	11,261–11,554	294	−7	ATT	TAA	-
trnT-aca	tRNA	J	11,604–11,670	67	49	-	-	TGT
trnP-cca	tRNA	N	11,677–11,743	67	6	-	-	GGT
nad6	pcg	J	11,831–12,400	570	87	ATG	TAA	-
cob	pcg	J	12,397–13,548	1152	−4	ATA	TAA	-
trnS2-tca	tRNA	J	13,572–13,637	66	23	-	-	AGT
trnL1-cta	tRNA	N	13,935–14,002	68	297	-	-	GAT
nad1	pcg	N	14,380–15,339	960	377	ATT	TAG	-
rrnL	rRNA	N	15,423–16,791	1369	83	-	-	-
trnV-gta	tRNA	N	16,860–16,925	66	68	-	-	CAT
rrnS	rRNA	N	16,926–17,696	771	0	-	-	-

## Data Availability

The mitochondrial genome has been deposited in the NCBI with accession number NC_079667.1.

## Supplementary Materials

The following supporting information can be downloaded at: https://www.mdpi.com/article/10.3390/cimb45120587/s1, Table S1: mitochondrial genomes of Vespidae used in the study; Table S2: the best partitioning scheme selected for ML analysis; Table S3: codon usage in the mitochondrial genome of *P. provsepa*.
