# Supplementary material for: Characterization of Complete Mitochondrial Genome and Phylogenetic Analysis of a Nocturnal Wasps—Provespa barthelemyi (Hymenoptera: Vespidae)
Source: Curr Issues Mol Biol. 2023 Nov 22;45(12):9368–77. doi: 10.3390/cimb45120587 (PMC10742571; doi:10.3390/cimb45120587)
Supplement: Supplementary file 1 [file cimb-45-00587-s001.zip › cimb-2698558-supplementary.pdf]

Table S1. Mitochondrial genomes of Vespidae species used in GenBank.

Subfamily	genus	Species	Genomes size (bp)	Accession Number
Vespinae	*Dolichovespula*	*Dolichovespula flora*	16,481	OP250139.2
		Dolichovespula lama	16,065	OP250140.1
		Dolichovespula panda	17,137	KY293679.1
		Dolichovespula saxonica	16,420	OP250141.1
		Dolichovespula xanthicincta	15,965	OP250142.1
		Dolichovespula kuami	15,671	NC_069159.1
	Vespa	*Vespa affinis*	19,109	NC_039134.1
		Vespa bicolor	16,937	KJ735511.1
		Vespa ducalis	15,779	KX950825.1
		Vespa magnifica	16,730	MT137097.2
		Vespa mandarinia	15,902	KR059904.1
		Vespa orientalis	16,101	KY563657.1
		Vespa simillima simillima	18,340	MN542756.1
		Vespa velutina	16,475	KY091645.1
		Vespa basalis	16,735	MK440075.1
	Vespula	*Vespula germanica*	16,342	KR703583.1
		Vespula flaviceps	17,489	NC_045215.1
		Vespula vulgaris	19,721	FR997693.1
		Vespula structor	16,475	NC_068892.1
	Provespa	*Provespa barthelemyi*	17,721	NC_079667.1
Polistinae	*Polistes*	*Polistes jokahamae*	16,616	KR052468.1

Table S2. Codon usage in the mitochondrial genome of *Provespa barthelemyi*

Codon	No.
ATT	376
TTA	360
TTT	342
ATA	273
AAT	176
TAT	142
AAA	121
TCT	112
TCA	108
GTT	94
CTT	81
ATC	79
GGA	78
TGA	75
AGA	69
ACA	69

TTC	68
TTG	64
GAA	61
CTA	60
CCT	55
GCT	53
CAT	53
GGT	50
CAA	50
ACT	50
GAT	45
AAC	45
ATG	44
GTA	43
TAC	42
GGG	34
CCA	33
TCC	29
TGT	26
CCC	26
AGT	25
AAG	22
GCA	20
CAC	18
CGA	18
GAG	16
CGT	16
ACC	16
CTC	13
GAC	11
GTG	11
TAA	11
TGG	10
AGG	9
GGC	7
GTC	7
GCC	6
TGC	6
CAG	6
CGG	6
TCG	4
AGC	3
CCG	1

TAG	1
GCG	1
CTG	0
CGC	0
ACG	0

Table S3. The best partitioning scheme selected by ModelFinder for ML analysis in Vespinae.

Model	LogL	AIC	w-AIC	AICc	w-AICc	BIC	w-BIC
GTR+F+I+G4	-100771.1883	201636.3766	1	201636.781	1	201980.61	1
TIM+F+I+G4	-100834.8485	201759.697	0	201760.068	0	202089.2822	0
TIM2+F+I+G4	-100855.0321	201800.0642	0	201800.4352	0	202129.6494	0
TVM+F+I+G4	-100873.877	201839.754	0	201840.1415	0	202176.6633	0
K3Pu+F+I+G4	-100931.6125	201951.225	0	201951.5798	0	202273.4861	0
TPM2+F+I+G4	-100975.3828	202038.7656	0	202039.1204	0	202361.0267	0
TPM2u+F+I+G4	-100975.4955	202038.991	0	202039.3458	0	202361.2521	0
GTR+F+G4	-100974.4016	202040.8032	0	202041.1907	0	202377.7125	0
TIM+F+G4	-101050.8617	202189.7234	0	202190.0782	0	202511.9845	0
TVM+F+G4	-101054.0052	202198.0104	0	202198.3814	0	202527.5956	0
TIM2+F+G4	-101076.1175	202240.235	0	202240.5898	0	202562.4961	0
TIM3+F+I+G4	-101074.5017	202239.0034	0	202239.3744	0	202568.5886	0
TN+F+I+G4	-101111.7712	202311.5424	0	202311.8972	0	202633.8035	0
K3Pu+F+G4	-101128.0801	202342.1602	0	202342.4992	0	202657.0972	0
TPM3+F+I+G4	-101141.5468	202371.0936	0	202371.4484	0	202693.3547	0
TPM3u+F+I+G4	-101141.5636	202371.1272	0	202371.482	0	202693.3883	0
TPM2u+F+G4	-101172.2784	202430.5568	0	202430.8958	0	202745.4938	0
TPM2+F+G4	-101172.2786	202430.5572	0	202430.8962	0	202745.4942	0
HKY+F+I+G4	-101193.5402	202473.0804	0	202473.4194	0	202788.0174	0
TIM3+F+G4	-101306.2786	202700.5572	0	202700.912	0	203022.8183	0
TPM3+F+G4	-101355.6497	202797.2994	0	202797.6384	0	203112.2364	0
TPM3u+F+G4	-101355.6511	202797.3022	0	202797.6412	0	203112.2392	0
TN+F+G4	-101362.1518	202810.3036	0	202810.6426	0	203125.2406	0
HKY+F+G4	-101426.2157	202936.4314	0	202936.755	0	203244.0442	0
GTR+F+I	-102235.2337	204562.4674	0	204562.8549	0	204899.3767	0
TVM+F+I	-102326.5921	204743.1842	0	204743.5552	0	205072.7694	0
TIM+F+I	-102354.3705	204796.741	0	204797.0958	0	205119.0021	0
TIM2+F+I	-102360.311	204808.622	0	204808.9768	0	205130.8831	0
TPM2u+F+I	-102448.3571	204982.7142	0	204983.0532	0	205297.6512	0
TPM2+F+I	-102448.4016	204982.8032	0	204983.1422	0	205297.7402	0
